# Canine peripheral blood mononuclear cell-derived B lymphocytes pretreated with lipopolysaccharide enhance the immunomodulatory effect through macrophage polarization

**DOI:** 10.1371/journal.pone.0256651

**Published:** 2021-11-22

**Authors:** Hee-Won Jang, Ju-Hyun An, Kyeong Bo Kim, Jeong-Hwa Lee, Ye-In Oh, Su-Min Park, Hyung-Kyu Chae, Hwa-Young Youn

**Affiliations:** Laboratory of Veterinary Internal Medicine, Department of Veterinary Clinical Science, College of Veterinary Medicine, Seoul National University, Seoul, Republic of Korea; National Institutes of Health, UNITED STATES

## Abstract

**Background:**

Preconditioning with lipopolysaccharide (LPS) is used to improve the secretion of anti-inflammatory agents in B cells. However, there are only a few studies on canine B cells.

**Objective:**

This study aimed to evaluate the immune regulatory capacity of canine peripheral blood mononuclear cell-derived B cells pretreated with LPS.

**Methods:**

Canine B cells were isolated from canine peripheral blood mononuclear cells, which were obtained from three healthy canine donors. The B cells were preconditioned with LPS, and then cell viability and the expression of the regulatory B cell marker were assessed. Finally, RNA extraction and immunofluorescence analysis were performed.

**Results:**

LPS primed B cells expressed the interleukin (IL)-10 surface marker and immunoregulatory gene expression, such as IL-10, programmed death-ligand 1, and transforming growth factor beta. Macrophages in the inflammatory condition cocultured with primed B cells were found to have significantly down-regulated pro-inflammatory cytokine, such as tumor necrosis factor-α, and up-regulated anti-inflammatory cytokines such as IL-10. Additionally, it was revealed that co-culture with primed B cells re-polarized M1 macrophages to M2 macrophages.

**Conclusions:**

This study revealed that LPS-primed B cells have an anti-inflammatory effect and can re-polarize macrophages, suggesting the possibility of using LPS-primed B cells as a therapeutic agent for its anti-inflammatory effects and immune modulation.

## Introduction

B cells perform several immunological functions, and because of their ability to produce antibodies, they have been primarily regarded as positive regulators of the immune response and central contributors to the development of immune-related diseases. In particular, it has been revealed by various studies that the anti-inflammatory effect of B cells is achieved by the expression of interleukin (IL)-10 [1, 2]. Moreover, there are multiple studies about relation between presence of B cells and reduced disease severity in autoimmune diseases [3, 4].

IL-10 is an anti-inflammatory cytokine that plays an important role in controlling the inflammatory response that causes tissue damage [5]. In addition, IL-10 plays an inhibitory role primarily by limiting the innate effector function of macrophages and dendritic cells and subsequent activation of T cells [6]. Therefore, B cells producing IL-10 are attracting attention as a pioneering cell therapy, and various studies are being conducted to apply IL-10-producing B cells as immunomodulatory treatments [7]. Therefore, to develop more effective cell therapy products, various studies are in progress to overexpress IL-10 in B cells [8, 9]. According to Parekh et al., when pre-stimulating B cells with lipopolysaccharide (LPS), the expression of IL-10 increased [10]. However, in order for LPS-primed B cells to be applied as a therapeutic agent to patients with inflammatory diseases and immune diseases, additional studies are needed to evaluate the mechanism by which IL-10-overexpressed B cells have anti-inflammatory effects.

Macrophage, which belongs to the innate immune system, is recognized to be involved in chronic inflammation, and it plays central roles in inflammatory diseases [11]. Many studies have been conducted to understand the mechanisms involved in activation of macrophage and to relate them to macrophage function. The well-known bipolar model [12] distinguishes macrophage into two main types of polarization: the classically activated type 1 macrophage (M1, pro-inflammatory type) and the alternatively activated type 2 macrophage (M2, anti-inflammatory type) [13]. Thus, in inflammatory disorders, down-regulating M1, or re-polarizing M1 to M2 are the two major approaches to relieve inflammation [14].

Preconditioning with LPS is used to improve the secretion of anti-inflammatory agents in B cells. However, there are only a few studies on canine B cells. Therefore, this study aimed to evaluate the anti-inflammatory effect of LPS-primed B cells from canines.

## Materials and methods

### B cell isolation

Canine B cells were isolated from canine peripheral blood mononuclear cells (cPBMCs). To isolate cPBMCs, all experimental procedures were approved by the Institutional Animal Care and Use Committee of Seoul National University (protocol no. SNU-201228-4). All protocols were in accordance with approved guidelines, and the article follows the Animal Research: Reporting of In Vivo Experiments guidelines. cPBMCs were collected using Ficoll-Paque PREMIUM (GE Healthcare Life Sciences, Uppsala, Sweden) according to the manufacturer’s instruction. With the owners’ consent, whole blood samples were obtained from three healthy canine donors. The blood samples were blended with an equal volume of Dulbecco phosphate buffered salt (DPBS) solution DPBS (Pan Biotech, Aidenbach, Germany) and placed on Ficoll-Paque PREMIUM according to the manufacturer’s guidelines.

B cells were isolated using mouse CD79a antibody (dilution, 1:200; Invitrogen, Carlsbad, CA, USA), anti-mouse immunoglobulin (Ig)-G microbeads (Miltenyi Biotec, Audurn, CA, USA) with MACS (Miltenyi Biotec, Audurn, CA, USA) according to the manufacturer’s instruction. A mixture of 10% fetal bovine serum (FBS) (Pan Biotech) and phosphate buffered saline (PBS) were used for the MACS separation buffer. The CD79a antibody was added to the cPBMCs, which were then incubated for 2 hours at 37°C in a humidified atmosphere with 5% carbon dioxide. The CD79a+ B cells were collected after centrifugation at 780 × *g* for 10 minutes. For magnetic labeling, 10 μl of anti-mouse IgG microbead and 40 μl of the MACS separation buffer were added to the CD79a+ B cells, which were incubated for 2 hours. The CD79a+ B cells were collected after centrifugation at 780 × *g* for 10 minutes. After washing the LS column of MACS with 3 mL of MACS separation buffer, the CD79a+ B cells were added to the LS column for isolation. After another washing with 3 mL of MACS separation buffer, the CD79a+ B cells were collected in 5 mL of MACS separation buffer. The B cell population obtained out of total cPBMCs were at the mean of 5%.

### Preconditioning B cells with lipopolysaccharide

To stimulate the B cells with LPS (Sigma-Aldrich, St. Louis, MO, USA), the cells were plated at a density of 1 × 10^5^ cells/mL in six-well plates. The medium used was the control, which did not contain LPS, and 5 ng/mL of LPS and 10 ng/mL of LPS were added to the experimental B cell groups. The cells were cultured for 24 hours for stimulation.

### Cell viability assay

Cell viability was assessed using the Cell Counting Kit-8 (CCK-8) assay (D-Plus^TM^ CCK cell viability assay kit; Dong-in Biotech, Seoul, Republic of Korea) to confirm that the concentrations of LPS were not cytotoxic for the B cells. The cells were seeded at a density of 5 × 10^5^ cells/well in a 96-well plate. The medium used was the control, which did not contain LPS, and 5 ng/mL of LPS and 10 ng/mL of LPS were added to the experimental B cell groups. After stimulating the cells for 24 hours, 10 μL of the Cell CCK solution was added, and the cells were incubated in the dark at 37°C for 1 hour. The absorbance at a 450-nm wavelength was determined with a spectrophotometer (Epoch Microplate Spectrophotometer; BioTek Instruments, Winooski, VT, USA).

### Flow cytometry analysis

We obtained LPS-primed B cells as described above, and then used the IL-10 antibody (dilution, 1:100; ABclonal, Woburn, MA, USA) to evaluate the expression of the regulatory B cell marker. After incubation for 1 hour, the cells were washed with DPBS. Indirect immunofluorescence was performed using mouse anti-rabbit IgG-PE (dilution, 1:100; Santa Cruz Biotechnology, Santa Cruz, CA, USA). Unstained cells were used as the negative control. Cell fluorescence was analyzed with a flow cytometer (FACS Aria II; BD Biosciences, Franklin Lakes, NJ, USA). The results were analyzed using FlowJo 7.6.5 software (Tree Star, Inc., Ashland, OR, USA).

### Western-blot analysis

Total proteins from cells were extracted using the PRO-PREP Protein Extraction Kit (iNtRON Biotechnology, Seongnam, South Korea) and measured using the Bio-Rad DC Protein Assay Kit (Bio-Rad Laboratories, Hercules, CA, USA). The total protein content in each 20 μg sample was subjected to SDS-PAGE and immunoblotting with antibodies against IL-10 (ABclonal Tech, MA, USA), TGF-β (Cusabio Biotech, Wuhan, China), PD-L1(Santa Cruz Biotechnology, CA, USA) and β-actin Santa Cruz Biotechnology, CA, USA).

### Co-culture system

RAW 264.7 and DH82 cells were purchased from the Korean Cell Line Bank (Seoul, Korea). Both cells were seeded in six-well plates at a density of 1 × 10^6^ cells/well and incubated overnight. After adherence to the plates was confirmed, 200 ng/mL of LPS was added for 24 hours. Next, the medium was removed, the cells were washed three times with DPBS and then replaced with the control medium. Using 0.4-μm pore size inserts, naïve and primed B cells were plated onto the macrophage cells at a density of 2 × 10^5^ cells/well and ratio of 5:1. The total number of cells were divided into four groups: macrophage cells at the bottom and no insert, macrophage in the inflammatory condition at the bottom and no inserts, macrophage in the inflammatory condition and naïve B cells in the upper chamber, and macrophage in the inflammatory condition and LPS-primed B cells in the upper chamber. All cells were incubated for 48 hours and then harvested for RNA extraction and immunofluorescence analysis.

### RNA extraction, complementary DNA synthesis, and real-time quantitative polymerase chain reaction

The Easy-BLUE Total RNA Extraction Kit (Intron Biotechnology, Seongnam, Korea) was used to isolate RNA according to the manufacturer’s instructions. For each sample, total RNA concentration was measured at 260-nm absorbance using a nanophotometer (IMPLEN, Munich, Germany). Complementary (c)-DNA was synthesized using Cell Script All-in-One 5× 1st cDNA Strand Synthesis Master Mix (Cell Safe, Seoul, Korea), and the samples were detected using AMPIGENE qPCR Green Mix Hi-ROX with SYBR Green dye (Enzo Life Sciences, Farmingdale, NY, USA) and forward and reverse primers (Bionics, Seoul, Korea). The expression levels of each gene were normalized to that of glyceraldehyde 3-phosphate dehydrogenase. The primer sequences used in this study are listed in Tables 1 and 2.

**Table 1:** Sequences of PCR mouse primers used in this study.

Gene	Forward (5’-3’)	Reverse (5’-3’)	Reference
mGAPDH	CAA AAT GGT GAA GGT CGG TG	CGT TGA TGG CAA CAA TCT CC	[15]
mIL-10	TGG CCC AGA AAT CAA GGA GC	CAG CAG ACT CAA TAC ACA CT	[16]
mTNF-α	GGC CTC TCA CCT TGT TGC C	ATG ACC CGT AGG GCG ATT AC	[17]
miNOS	CAC CTT GGA GTT CAC CCA GT	AGA TGT AGG TTA TTT TCT GCC AGT G	[18]
mCD206	AAC GGA ATG ATT GTG TAG TTC TAG C	TAC AGG ATC AAT AAT TTT TGG CAT T	[19]

**Table 2:** Sequences of canine PCR primers used in this study.

Gene	Forward (5’-3’)	Reverse (5’-3’)	Reference
cGAPDH	CCC CAA TGT ATC ACT TGT GGA TCT G	CCT GCT TCA CTA CCT TCT TGA TGT C	[20]
cIL-10	AGC ACC CTA CTT GAG GAC GA	GAT GTC TGG GTC GTG GTT CT	[21]
cTNF-α	CCA AAC CGA CCC TTT GAT CA	CCA GCC CTG AGC CCT TAA TT	[22]
ciNOS	GAG ATC AAT GTC GCT GTA CTC C	TGA TGG TCA CAT TTT GCT TCT G	[16]
cCD206	GGA AAT ATG TAA ACA GGA ATG ATG C	TCC ATC CAA ATA AAC TTT TTA TCC A	[16]
cPD-L1	CCG CCA GCA GGT CAC TT	TCC ATT GTC ACA TTG CCA CC	[23]
cTGF-β	CTC AGT GCC CAC TGT TCC TG	TCC GTG GAG CTG AAG CAG TA	[24]

### Immunofluorescence analysis

RAW 264.7 and DH82 cells were washed three times with DPBS and fixed with 4% paraformaldehyde for 20 minutes at room temperature. After washing with DPBS, the cells were permeabilized for 1 hour with 0.2% Triton X-100 (Sigma-Aldrich) and then blocked for 1 hour at room temperature with 2% FBS. The cells were incubated sequentially with FITC-conjugated CD206 (1:200; Santa Cruz Biotechnology) and PE-conjugated CD11b antibodies (1:200; Abcam, Cambridge, MA, USA) at 4°C overnight in the dark. Finally, the cells were washed three times with PBS and mounted. The samples were observed using an EVOS FL microscope (Life Technologies, Darmstadt, Germany). Immunoreactive cells were counted in 20 random fields per group, and the percentage of CD206+ positive cells was recorded.

### Statistical analysis

GraphPad Prism version 6.01 software (GraphPad Software, La Jolla, CA, USA) was used to perform the statistical analysis. The Student t-test and one-way analysis of variance were used to analyze the data, followed by the Bonferroni multiple comparison test. The data are presented as mean value ± standard deviation. Differences with a P-value <0.05 were considered statistically significant.

## Results

### Lipopolysaccharide-stimulated B cells

**Fig 1:**
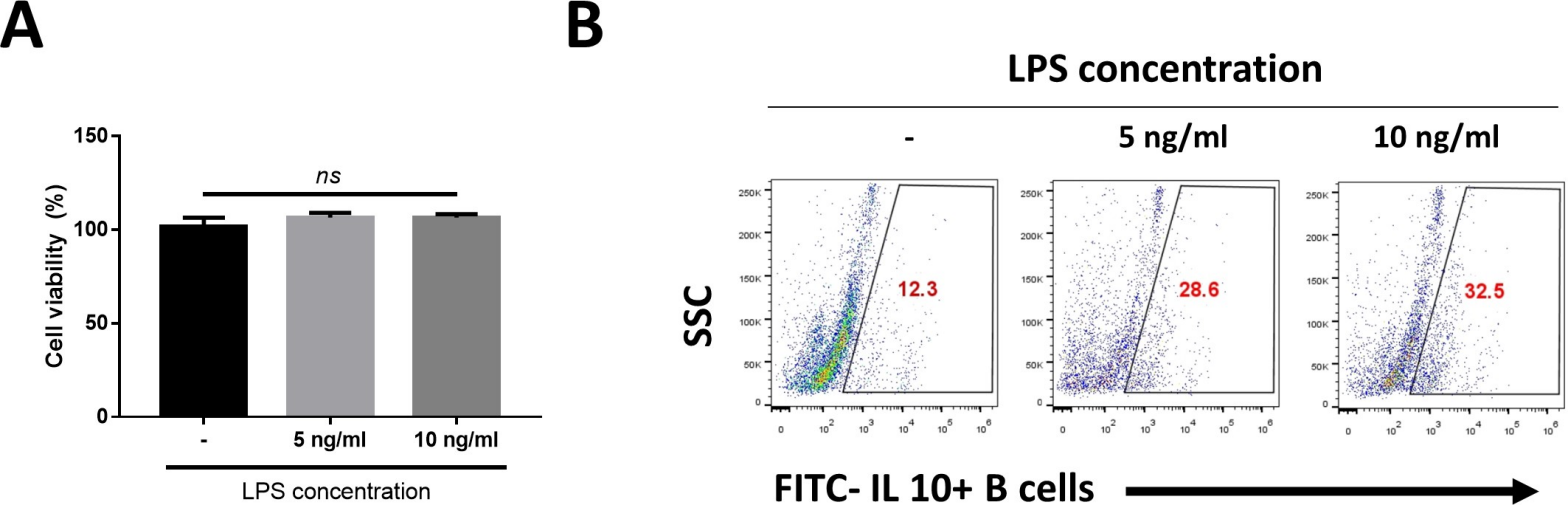
Characterization of B cell isolated from canine peripheral blood mononuclear cells. (A) Cell viability assay. Cell viability of B cells according to the LPS concentration gradient was confirmed through CCK-8 analysis. (B) Through flow cytometry, the degree of increase in B cells expressing IL-10 was confirmed. Representative of three independent experiments with similar results. Data are shown as mean ± standard deviation of three independent experiments (*ns* = Not Statistically Significant by one-way ANOVA analysis).

**Fig 2:**
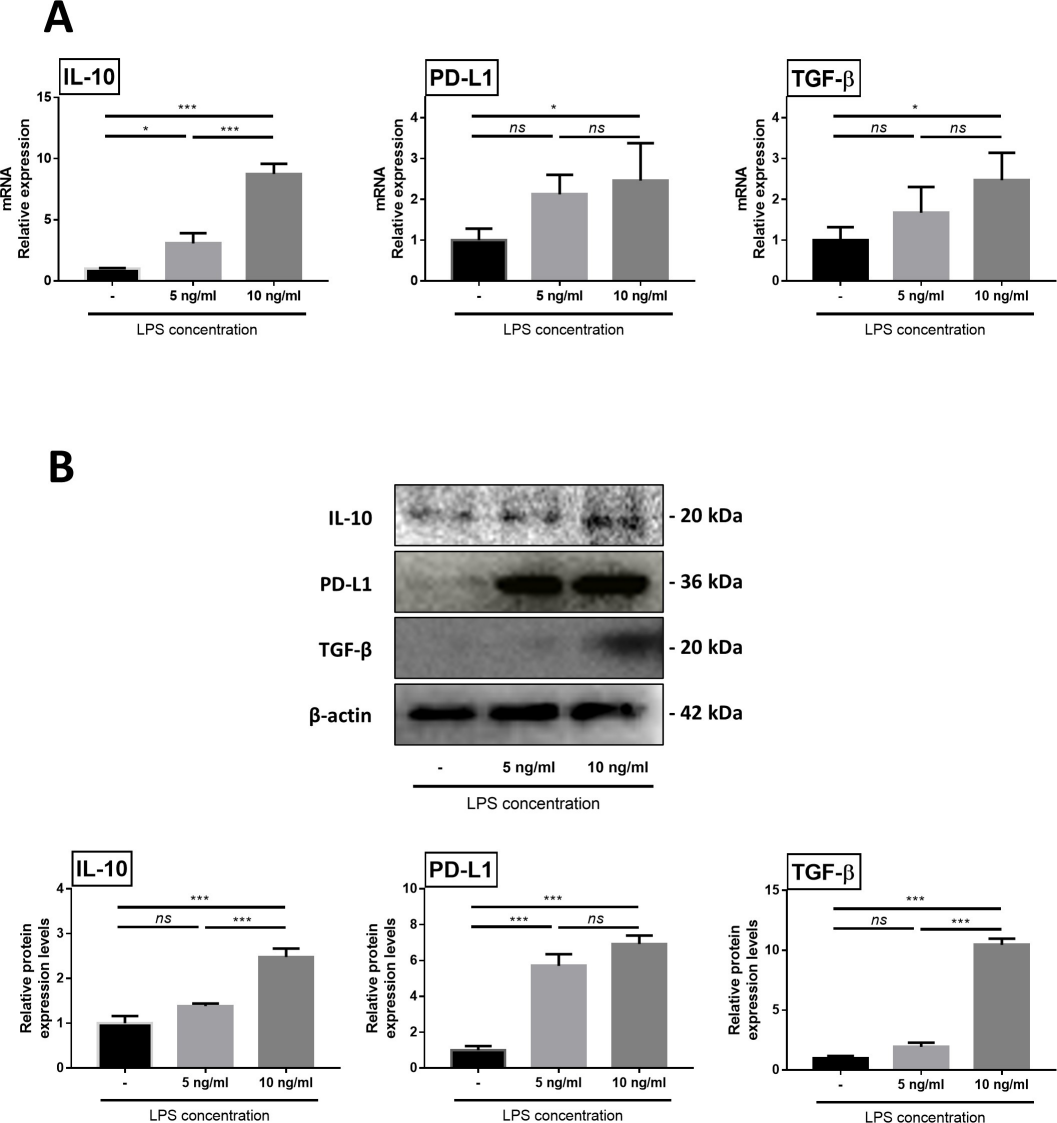
Expression of immunoregulatory factor. (A) Increased gene expression of IL-10, PD-L1 and TGF-β in primed B cells. (B) Increased protein expression of IL-10, PD-L1 and TGF-β in primed B cells. Results are presented as the mean ± standard deviation of three independent experiments. Data are shown as mean ± standard deviation of three independent experiments (*ns* = Not Statistically Significant, *P < 0.05, **P < 0.01, ***P < 0.001 by one-way ANOVA analysis).

B cells were obtained with MACS positive selection kit using CD79a as antibody. Considering efficiency of B cell isolation kits from MACS, exception of cell debris and platelet, B cell purity would be over 90% out of lymphocyte population [25]. We used the CCK-8 assay to evaluate the cytotoxicity of 5 ng/mL of LPS and 10 ng/mL LPS to B cells. The result showed there was no significant difference between the control and experimental groups (Fig 1A). After stimulation with LPS, we determined that both 5-ng/mL-LPS and 10-ng/mL LPS-stimulated B cell groups expressed IL-10, which is known as a regulatory B cell expression marker, based on flow cytometry analysis (Fig 1B). In addition, both groups expressed increased immunomodulatory factors, such as IL-10, programmed death-ligand 1 (PD-L1), and transforming growth factor beta (TGF-β), and this result was more significant in the 10 ng/mL-LPS-stimulated group (Fig 2A). In addition, western blot analysis was performed to confirm protein expression, and it was confirmed that there was a similar tendency to expression of mRNA (Fig 2B). Since the effects of LPS occurred in a dose-dependent manner, we chose the 10-ng/mL-LPS primed B cells for further study.

### Anti-inflammatory effect of the primed B cells

**Fig 3:**
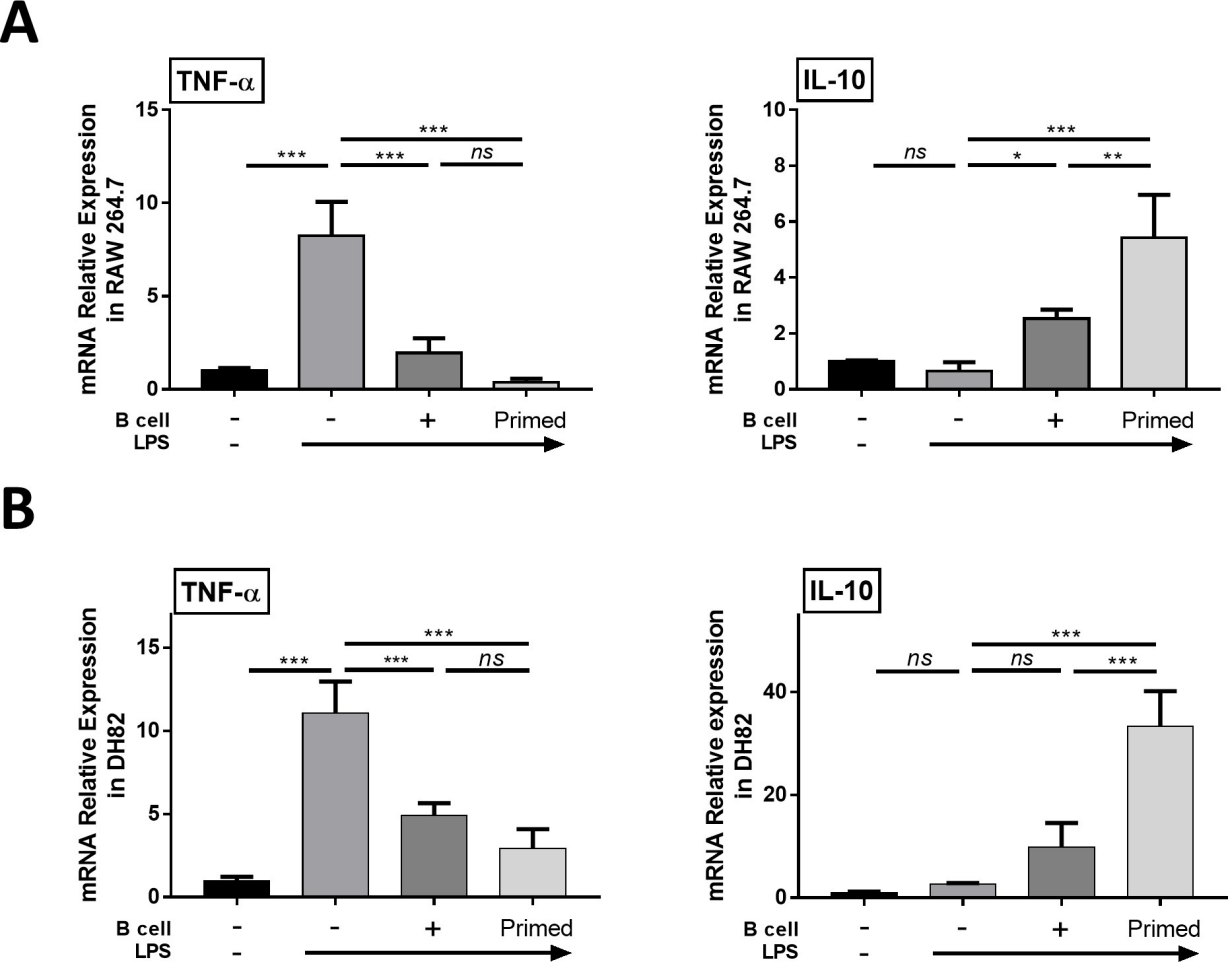
Changed in mRNA expression of inflammation. LPS stimulated RAW 264.7 and DH82 cells were co-cultured with primed B cells for 48 h. (A) Relative mRNA expression levels of TNF-α and IL-10 in RAW 264.7. (B) Relative mRNA expression level of TNF-α and IL-10 in DH82. Data are shown as mean ± standard deviation of three independent experiments (*ns* = Not Statistically Significant, *P <0.05, **P <0.01, ***P < 0.001 by one-way ANOVA analysis).

The result of RT-qPCR showed that after LPS stimulation, TNF-α expression levels were highly increased, and they were decreased after co-culture in both the naïve and LPS-primed B cell groups. This result was more significant in the co-cultured with LPS-primed B cell group than in the naïve B cell group. Moreover, the level of anti-inflammatory cytokine IL-10 expression was increased after co-culture in both B cell groups, and this result was more significant after co-culture in the LPS-primed B cell group than in the naïve B cell group. All these results were observed in the RAW 264.7 and DH82 cell lines (Fig 3A and 3B). Therefore, these results indicated that primed B cells have anti-inflammatory effects by macrophage polarization.

### Macrophage polarization from M1 to M2 like phenotype

**Fig 4:**
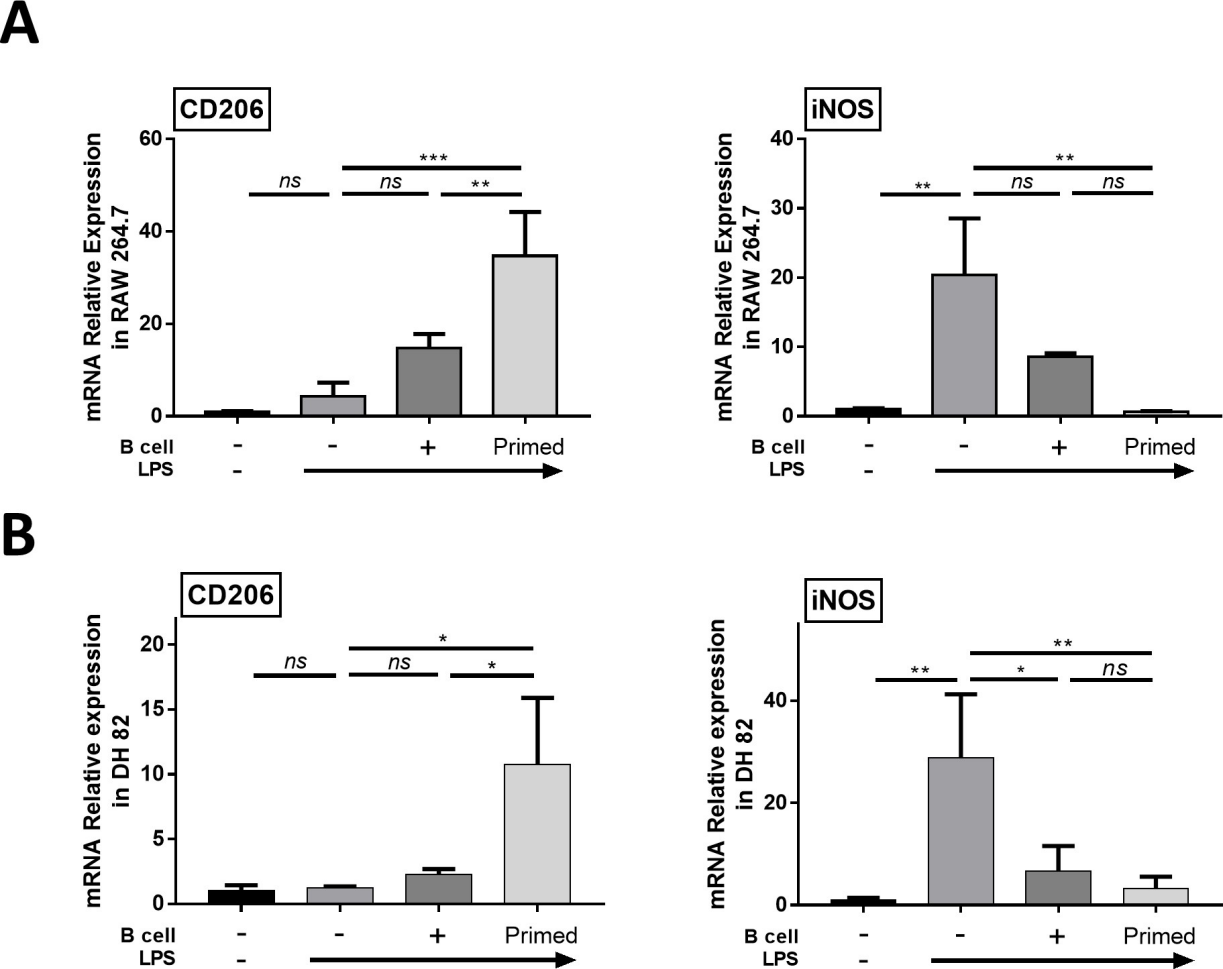
Primed B cells induced the expression of M2 macrophage markers in RAW 264.7 cells and DH82 cells. LPS stimulated RAW 264.7 and DH82 cells were co-cultured with primed B cells for 48 h. (A) Relative mRNA expression levels of CD206 and iNOS in RAW 264.7. (B) Relative mRNA expression level of CD206 and iNOS in DH82. Data are shown as mean ± standard deviation of three independent experiments (*ns* = Not Statistically Significant, *P < 0.05, **P < 0.01, ***P < 0.001 by one-way ANOVA analysis).

**Fig 5:**
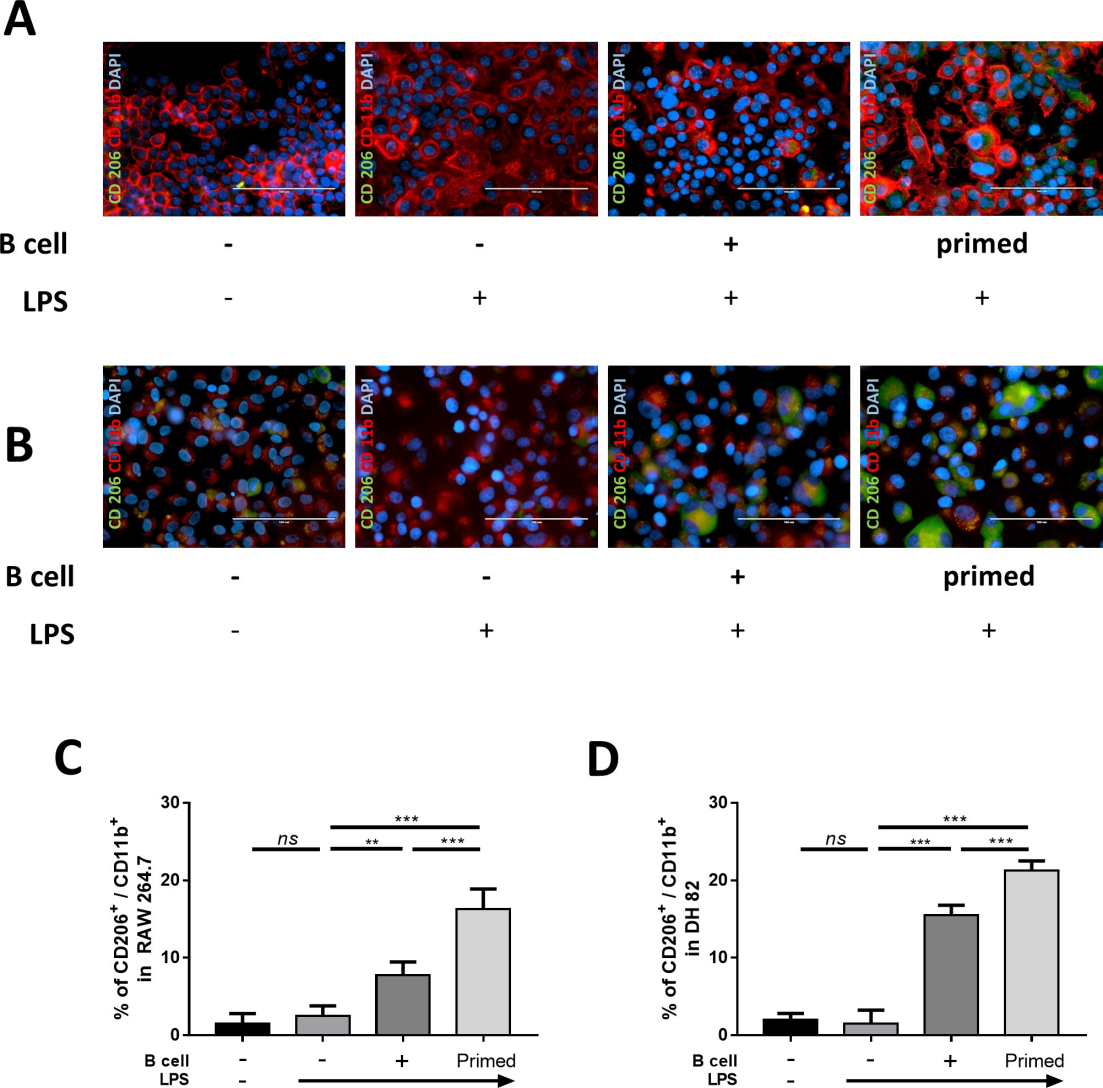
Primed B cells induced the expression of M2 macrophage in RAW 264.7 cells and DH82 cells. LPS stimulated RAW 264.7 and DH82 cells were co-cultured with primed B cells for 48 h (Scale bar, 100um). (A) Representative immunofluorescence staining using anti-CD11b-PE or anti-CD206-FITC positive cell in RAW 264.7 cells (B) Representative immunofluorescence staining using anti-CD11b-PE or anti-CD206-FITC positive cell in DH82 cells (C) The calculated percentage of CD206-FITC positive cells among the CD11b-PE positive cell in RAW 264.7 are shown (D) The calculated percentage of CD206-FITC positive cells among the CD11b-PE positive cell in DH82 are shown. Data are shown as mean ± standard deviation of three independent experiments (*ns* = Not Statistically Significant, *P < 0.05, **P < 0.01, ***P < 0.001 by one-way ANOVA analysis).

We evaluated *CD206* as the M2 macrophage target gene and *iNOS* as the M1 macrophage target gene in RAW 264.7 and DH82 cells for RT-PCR (Fig 4A and 4B). The results showed that *iNOS* expressions were highly increased after LPS stimulation, and they were decreased after co-culture with both naïve and LPS-primed B cells; this result was more significant in the LPS-primed B cell co-culture group than in the naïve B cell co-culture group. For immunofluorescence assay, we used CD206 as M2 macrophage marker protein. The results showed that in both RAW 264.7 and DH82 cells, the percentage of CD206+ M2 macrophages was increased in both naïve and LPS-primed B cell co-cultured groups, and this finding was more significant in the LPS-primed B cell group than in the naïve B cell co-culture group (Fig 5A and 5B). These results suggest that B cells would have an ability to repolarize the M1 like macrophage to the M2 like macrophage, and this ability is enhanced considerably when B cells are primed with LPS.

## Discussion

Cell therapy is the most recent phase of biotechnology in medicine, and it offers the advantage of treating and altering the course of diseases, which cannot be addressed by current pharmaceutical techniques [26]. B cells are generally considered to play a pathogenic role in the development of autoimmune diseases because they produce autoantibodies that cause target tissue damage, but autoantibodies can also exert a protective effect through the elimination of apoptotic cells and reduction of autoantigen load [27]. B cells also act as antigen-presenting cells that contribute to the activation and amplification of naive and activated autoreactive T cell responses [8]. However, in order to apply B cells as cell therapy to patients with various autoimmune diseases, including rheumatoid arthritis, autoimmune diabetes, autoimmune encephalomyelitis and lupus, additional studies about the treatment mechanisms are needed [3].

LPS, which a major component of environmental microbial products, is one of the most well-studied pathogen-associated molecular patterns that can induce innate immune recognition. It signals through the toll-like receptor 4 (TLR-4) [28], and interacts with dendritic cells, macrophages, and B cells [29–32]. In B cells, activation of pattern recognition receptors, especially members of the toll-like receptor family, has been shown to be an effective stimulus to induce IL-10 production. In these cells, stimulation of TLR-4 results in transcriptional activation of the IL-10 gene, increasing IL-10 protein production and secretion. According to Xu et al., when B cells were pre-stimulated with LPS, the expression of IL-10 was increased [33]. Our study confirmed that the number of cells expressing IL-10 increased when B cells were pretreated with LPS [9]. In addition, LPS-primed B cells expressed regulatory B cell markers, such as IL-10, PD-L1, and TGF-β [34, 35].

B cells that produce IL-10 and express TGF-β and PDL-1 are known as regulatory B cells (Bregs) in the B cell subset [34, 36, 37], and Bregs have the effect of suppressing activated immunity [7]. Therefore, various studies are being conducted with the goal of applying B cells as therapeutic agents to patients with immune-related inflammatory diseases. However, there has been no research on this in veterinary medicine. However, in order to apply IL-10-overexpressing B cells as a therapeutic agent to patients with immune-related inflammatory diseases, it is necessary to determine how IL-10-overexpressing B cells regulate immune cells in inflammatory conditions.

Macrophages are being used to confirm the immunomodulatory effect in various disease models. In the inflammatory condition, M1 is responsible for active inflammation, such as the expression of high levels of pro-inflammatory, enhanced phagocytosis and assistance with T-helper type 1 cells, whereas M2 is responsible for immune modulation and wound repair functions [38]. Currently, strategies to reduce M1 or re-polarization of M1 to M2 have been studied, and re-educating M1 to M2 can be beneficial in not only decreasing pro-inflammatory effects but also inducing anti-inflammatory effects as well. Furthermore, there are studies about the requirement of IL-10 for the macrophage M2 like polarization [39, 40]. In the present study, RAW 264.7 and DH82 macrophages in inflammatory conditions were co-cultured with LPS-primed B cells to evaluate their effect on re-polarizing M1 to M2 [41, 42]. Other studies have shown shift in macrophage polarization from M1 to M2 phenotype by iNOS and CD206 respectively [43, 44], and it was accompanied by diminished TNF-α and elevated IL-10 levels where more cells were positive for iNOS [43, 45], which are corresponding with our study. As a result, it was confirmed that the polarization of macrophages for M2 like phenotype was greater when cells were co-cultured with B cells primed with LPS, which express higher IL-10 than control. Considering that changing macrophages from M1 to M2 in various inflammatory diseases is a major therapeutic target to suppress activated immunity, our results are considered to be the main supporting data for applying primed B cell as an immunotherapy. In addition, not only murine but also canine-derived macrophage cell lines were used in this experiment, and the test results will serve as important basic data that can be applied not only to future experimental models but also to patients with naturally occurring inflammatory diseases.

This study had a limitation in that it was not possible to determine which of the overexpressed factors were key when B cells were pretreated with LPS. Previous studies have been conducted on the immunomodulatory effect of B cells. Khan dt al explained that the immunotherapeutic ability of these Breg cells was demonstrated to be PD-L1 dependent. In this study, high PD-L1 expression in B cells defines a potent suppressive mechanism of humoral immunity through regulation of T cells. Also, as a result of this study, it was suggested that increasing the expression of PD-L1 in B cells could be a means to achieve the effect of enhancing immunomodulation. In addition, Scapini et al suggest that IL10–producing B cells plays an important role in suppressing inflammation and establishing anti-inflammatory networks that are crucial to maintain the balance between pathogenic T-cell subsets and beneficial Treg cells in inflammation associated with SLE-like disease. Moreover, Kessel et al demonstrated that TGF-beta released by B cells is a key factor in the immunomodulatory effect of B cells and has inhibitory efficacy in activated immune responses. Although it is not clear whether there are any major factors that enhance the effectiveness of primed B cells in this study, the factors revealed here were found to be significantly increased when B cells were pretreated with LPS. In addition, as a result of incubation with these pretreated B cells, it was confirmed that macrophages were effectively transformed into an anti-inflammatory type such as M2 type. These studies are expected to serve as important basic data for the application of immune cell therapeutics to inflammatory disease models in the future.

In conclusion, it was confirmed that the expression of IL-10 increased when B cells were pretreated with LPS. In addition, our data suggest that IL-10-overexpressed B cells play an important role in suppressing inflammation though macrophage polarization from M1 to M2. This study could serve as a basis for future in vivo studies on the anti-inflammatory effects of LPS-primed B cells, and clinical applications of canine B cells may become a new option of cell therapy for refractory inflammatory diseases.

## Supporting information

S1 Fig Protein expression of IL-10, PD-L1 and TGF-β in primed B cells in Fig 2. (TIF)
