# Supplementary figures and images for: Canine peripheral blood mononuclear cell-derived B lymphocytes pretreated with lipopolysaccharide enhance the immunomodulatory effect through macrophage polarization
Source: PLoS One. 2021 Nov 22;16(11):e0256651. doi: 10.1371/journal.pone.0256651 (PMC8608335; doi:10.1371/journal.pone.0256651)

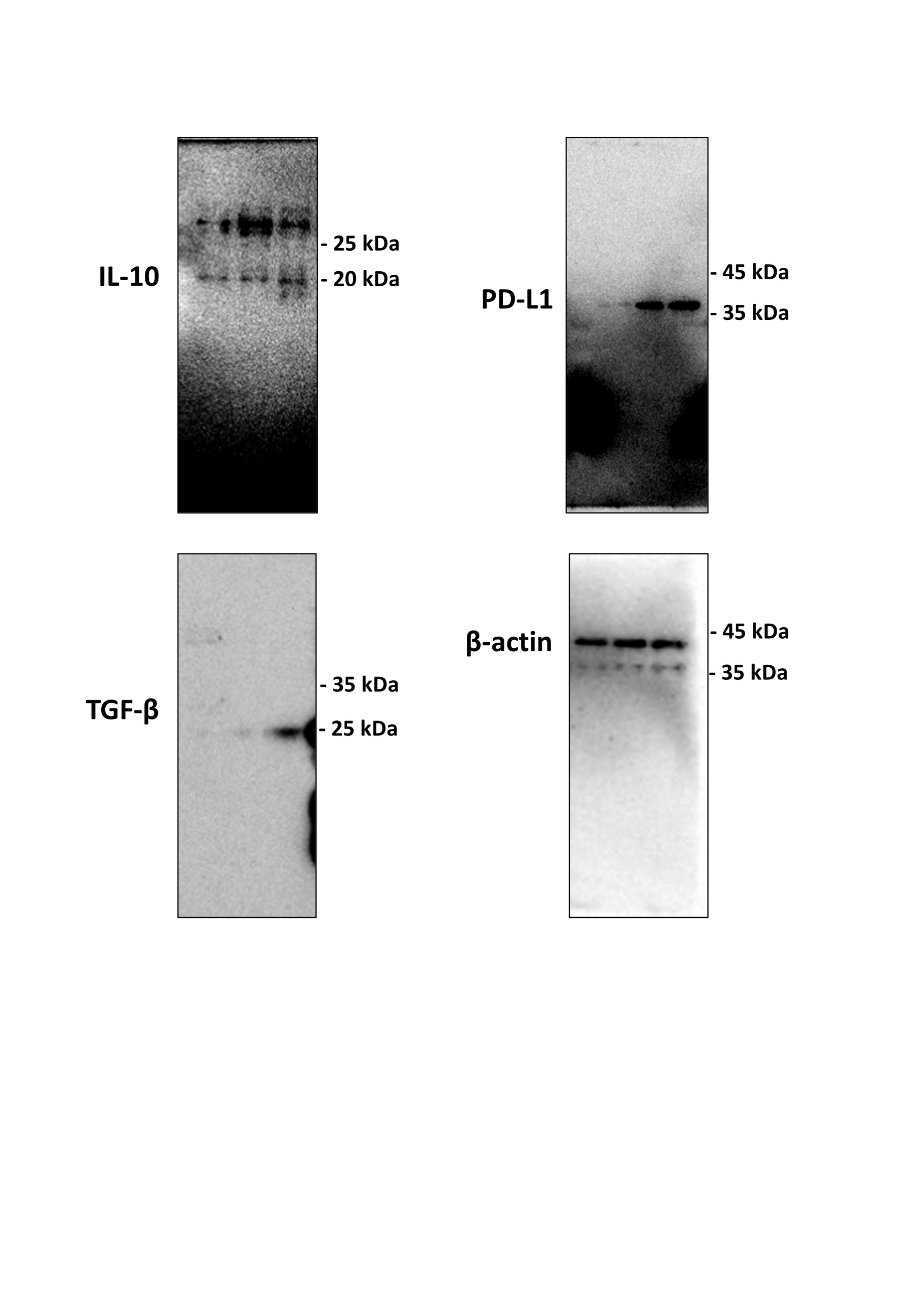

Supplement: S1 Fig — (TIF) [file pone.0256651.s001.tif]
